# Targeted Degradation of Androgen Receptor by VNPP433-3β in Castration-Resistant Prostate Cancer Cells Implicates Interaction with E3 Ligase MDM2 Resulting in Ubiquitin-Proteasomal Degradation

**DOI:** 10.3390/cancers15041198

**Published:** 2023-02-14

**Authors:** Elizabeth Thomas, Retheesh S. Thankan, Puranik Purushottamachar, David J. Weber, Vincent C. O. Njar

**Affiliations:** 1 Department of Pharmacology, University of Maryland School of Medicine, 685 West Baltimore Street, Baltimore, MD 21201, USA; 2 The Center for Biomolecular Therapeutics, University of Maryland School of Medicine, 685 West Baltimore Street, Baltimore, MD 21201, USA; 3 Isoprene Pharmaceuticals, Inc., 801 W Baltimore Street, Suite 502J, Baltimore, MD 21201, USA; 4 Department of Biochemistry and Molecular Biology, University of Maryland School of Medicine, Baltimore, MD 21201, USA; 5 Marlene and Stewart Greenebaum Comprehensive Cancer Center, University of Maryland School of Medicine, 685 West Baltimore Street, Baltimore, MD 21201, USA

**Keywords:** molecular glue, androgen receptor (f-AR/AR-V7) degrader, prostate cancer, HiBiT CRISPR cell, targeted protein degradation

## Abstract

**Simple Summary:**

In this study, we demonstrate the potential role of VNPP433-3β as molecular glue that induces physical proximity between the Androgen Receptor and MDM2 E3 ligase in prostate cancer cells. The interaction promotes MDM2-mediated ubiquitination of AR and its subsequent proteasomal degradation resulting in growth inhibition of prostate cancer cells.

**Abstract:**

Targeted protein degradation is a fast-evolving therapeutic strategy to target even the traditionally undruggable target proteins. Contrary to the traditional small-molecule inhibitors of enzyme or receptor antagonists that bind the active site pockets in the target protein, molecular glue degraders facilitate interaction of target proteins with E3 ubiquitin ligases by stabilizing the ternary complex and induce physical proximity, thereby triggering ubiquitination and subsequent proteasomal degradation. AR plays a key role in all stages of prostate cancer. It is activated by the binding of androgenic hormones and transcriptionally regulates multiple genes including the ones that regulate cell cycle. Using HiBiT CRISPR cell line, biochemical methods, and RNA sequencing, we report the potential role of VNPP433-3β, the next generation galeterone analog as molecular glue that brings together AR, the key driver of prostate cancer and MDM2, an E3 ubiquitin ligase leading to ubiquitination and subsequent degradation of f-AR and AR-V7 in prostate cancer cells.

## 1. Introduction

Targeted protein degradation (TPD) is a much-celebrated approach in today’s drug discovery efforts. Lately, close to 15 targeted degraders ranging from heterobifunctional PROTACs to molecular glues are expected to reach the patients soon [1]. Physical proximity of participating biomolecules plays a crucial role in virtually all biochemical processes including cell signaling, protein synthesis and degradation, the target pathways of most cancer drugs [2]. PROTAC (proteolysis targeting chimera) is a heterobifunctional small molecule with two active domains separated by a linker wherein one domain specifically binds the target protein to be degraded and the other interacts with an E3 ubiquitin ligase, bringing both the partners in proximity for ubiquitination and subsequent proteasomal degradation. Distinctly, molecular glues are chemical inducers of proximity thereby promoting dimerization or co-localization of partner proteins by forming a ternary complex and achieving the biochemical goal [3,4]. This class of molecules promotes protein-protein interactions and stabilizes the ternary complex by directly interacting with both partner proteins, resulting in various biological and pharmacological effects primarily due to inducing proximity. Among the several merits of molecular glue degraders over the conventional small-molecule enzyme inhibitors or receptor antagonists are their substochiometric level required to achieve the pharmaceutical goal, ability to target traditionally undruggable proteins and high specificity combined with potentially low off-targets. While the traditional enzyme inhibitors and receptor antagonists bind the active site pocket of the target protein, molecular glue small molecules induce and stabilize the interaction between two protein molecules, particularly the target proteins with ubiquitin ligases [4].

Androgenic hormone-regulated transcriptional activity of androgen receptor (AR) is vital for the normal development and function of the prostate but aberrant AR signaling is a major hallmark and driver of prostate cancer, the second highest cancer of men in the US and worldwide [5]. Castration-resistant prostate cancer (CRPC) is the advanced stage of cancer wherein AR is still a key player. Few small-molecule molecular glue degraders including PROTAC that selectively target AR and mediates its degradation are reviewed [6,7,8,9,10]. VNPP433-3β is a lead next generation galeterone analog that binds AR and promotes its degradation in prostate cancer cell lines and in vivo models of prostate cancer including AR-overexpressing PCa xenograft [11,12,13]. Further, we have shown that VNPP433-3β downregulates the transcription of EMT and cancer stem cell genes by selectively degrading the receptor transcriptional factor AR [14]. Additionally, it promotes the degradation of Mnk1/2 thereby decreasing phosphorylation of eIF4E and decreases 5′-cap-mediated translation [13]. Classically, AR plays a critical role in the development and progression of all stages of prostate cancer and remains a major therapeutic target [15]. Aberrant expression and or activity of AR is observed in most clinical cases of prostate cancer and is directly correlated to poor survival of patients [15].

In this study, using biochemical, molecular biology, and CRISPR-edited 22Rv1 HiBiT cell line that combines the power of CRISPR-mediated gene editing and bioluminescent reporter system, we demonstrate the molecular glue degrader role of VNPP433-3β that chemically induces proximity and brings together the target f-AR and E3 ubiquitin ligase MDM2, resulting in ubiquitination and subsequent proteasomal degradation of f-AR. Our findings of VNPP433-3β as a molecular glue degrader of f-AR/AR-V7 further reiterate the potential development of VNPP433-3β for treating advanced CRPC.

## 2. Materials and Methods

### 2.1. Cell Culture and Reagents

Prostate cancer cell lines, CWR-R1, CWR22Rv1 and LNCaP were procured from ATCC (Manassas, VA, USA) and cultured as recommended in RPMI-1640 media containing 10% heat-inactivated fetal bovine serum (FBS, GIBCO) and 1% penicillin-streptomycin (10,000 U/mL, Life Technologies, Carlsbad, CA, USA) at 37 °C and 5% CO_2_. Chemical synthesis of Galeterone (Gal) and VNPP433-3β was performed as described earlier and dissolved in cell culture-grade DMSO [12]. The primary antibodies targeting human AR, Mnk1, Mnk2, GAPDH, β-actin, α-tubulin, Histone H3, Ubiquitin and secondary HRP-conjugated anti-rabbit and anti-mouse used in the study were procured from Cell Signaling Technology, USA. Dihydrotestosterone (DHT), MG132 and all fine chemicals were purchased from Sigma Aldrich, St. Louis, MI, USA.

### 2.2. Immunoblotting

The cells were harvested, washed in PBS, and lysed in RIPA buffer containing 1× protease inhibitors (Roche, Indianapolis, IN, USA), phosphatase inhibitors (Thermo Scientific, Waltham, MA, USA) and 1 mmol/L EDTA and 1 mmol/L PMSF (Sigma, Kawasaki, Japan). Immunoblotting was carried out as described earlier [16]

### 2.3. Preparation of Nuclear and Cytoplasmic Fractions

Nuclei were isolated from the cultured cells that were treated with the VNPP433-3β or androgen (DHT) using NE-PER Nuclear and Cytoplasmic Extraction Kit (Thermo Fisher Scientific, Waltham, MA, USA) following manufacturer’s instructions as previously described [17]. Subsequently, equal amounts of protein were separated in SDS-PAGE and immunoblotted for AR and organelle-specific markers α-tubulin (cytoplasmic marker) and Histone H3 (nuclear marker).

### 2.4. Molecular Docking

The crystalline structure of human E3 ligase MDM2 (amino acids 17–125) available in the protein databank PDB (PDB id 2AXI) was employed for docking the ligand VNPP433-3β using the algorithm Autodock VINA 1.1.2. The co-crystallized ligand and water molecules were removed and polar Hydrogens and Kollman charges were added to the protein prior to docking as described previously [18]. VNPP433-3β was docked to the protein, centering the grid box at 5.599, 11.626 and 3.978 for x, y and z respectively with grid points of 60 each in x, y and z dimensions and default spacing. Rigid docking was carried out with exhaustiveness of conformational sampling at eight and other parameters at default. Out of nine poses generated by VINA, the binding pose with the least Gibb’s free energy of binding (ΔG°) and thus the highest affinity was selected, and the simulations were processed in BIOVIA Discovery Studio Visualizer v21.1.0.20298, Dassault Systemes, San Diego, CA, USA.

### 2.5. Co-Immunoprecipitation (Co-IP) Assay

CWR-R1 cells were hormone-starved for 48 h, treated with 10 µM VNPP433-3β for 4 h and lysed with lysis buffer (25 mM Tris-HCl pH 7.4, 150 mM NaCl, 1% NP-40, 1 mM EDTA, 5% glycerol). Immunoprecipitation of full-length AR (fAR) was carried out using Dyna beads conjugated to Protein A following manufacturer’s instructions (Thermo Fisher Scientific, Waltham, MA, USA). Dyna beads conjugate was incubated with 3 μg antibody and the Dyna beads-Ab complex was washed in washing buffer and successively in binding buffer. The cell lysate of 1 mg protein was then incubated with Dyna beads-Ab complex for 10 min. The complex was resuspended in elution buffer after washing in washing buffer and immunoblotted. The blot was probed with antibodies anti-fAR and anti-MDM2. IgG served as negative control.

### 2.6. Real Time Quantitative Studies of AR Degradation in CRISPR Knock-In HiBiT Reporter Cell Line

CRISPR-edited 22Rv1 cells that express endogenous AR labeled with HiBiT, a small 11 amino acid peptide is obtained from Promega Corp, Madison, WI, USA (Item # CS3023312; AR-HiBiT KI 22Rv1 CPM), grown in RPMI medium (Gibco, Waltham, MA, USA) supplemented with 10% FBS (Gibco) and 1× PenStrep (Gibco) in T25 cell culture flasks and assayed as described previously [19]. Briefly, the cells were transfected with LgBiT expression vector (Promega, #N2681) using FuGENE transfection reagent (Promega #E5911) according to the manufacturer’s instructions. Next day, 20,000 transfected cells were transferred to each well of the 96-well white culture plates (CoStar, Washington, DC, USA). The second day, the media was replaced by 1× solution of Endurazine (Nano-Glo^®^ Endurazine™ Promega # N2570) prepared in CO_2_-independent medium (Gibco # 18045088). The plates were incubated at 37 °C and 5% CO_2_ for 2.5 h to allow luminescence to equilibrate. Specified concentration of VNPP433-3β was added to five wells each, luminescence was read every 10 min for 5 h in TECAN Spark multimode microplate reader (Tecan Group Ltd., Männedorf, Switzerland). Relative luminescence unit (RLU) was plotted against time using Graphpad Prism (La Jolla, CA, USA).

### 2.7. RNA-Sequencing and Transcriptome Analyses

Total RNA was isolated from 22Rv1 cells treated with 10 μM VNPP433-3β for 24 h in triplicates using RNAeasy Plus mini kit (Qiagen, Hilden, Germany). The RNA was quantified, and the quality of preparation was assessed using Agilent 2100 Bioanalyzer. Only preparations with RNA Integrity Number (RIN) 8 or above was considered for the preparation of library using NEB Ultra II Directional RNA library prep kit. The libraries were quality-checked using Qubit and Agilent 2100 Bioanalyzer. RNA sequencing was carried out in Illumina NovaSeq S2 PE100 bp lane at Maryland Genomics, Institute for Genome Sciences, University of Maryland Baltimore. The quality of sequencing was measured by Phred quality score (Q score). Differential gene expression and Gene Set Enrichment analyses (GSEA) were carried out to reveal the genes and cellular metabolic and signaling pathways modulated by VNPP433-3β.

### 2.8. Statistical Analysis

Statistical analysis of differentially expressed genes were carried out and volcano plot was generated using GSEA (Broad Institute, Cambridge, MA, USA) and GraphPad Prism 9. A probability value with * *p* < 0.05, ** *p* < 0.01 and *** *p* < 0.001 were considered statistically significant. All experiments were performed three times independently.

## 3. Results

### 3.1. VNPP433-3β Induces Targeted Degradation of AR/AR-V7

**Figure 1:**
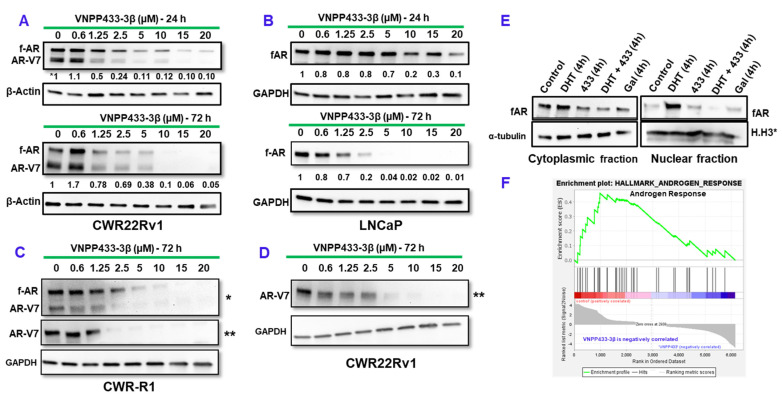
VNPP433-3β causes degradation of f-AR in a concentration-dependent manner. (**A**–**D**) Immunoblots of f-AR and AR-V7 following treatment with varying concentrations of VNPP433-3β (433) for 24 or 72 h in CWR22Rv1, LNCaP and CWR-R1 cells. * Probed with f-AR antibody and ** AR-V7-specific antibody. GAPDH or β-actin served as loading control. (**E**) Nuclear translocation of f-AR is inhibited by VNPP433-3β (433). 22Rv1 cells were treated with the androgen dihydrotestosterone (DHT), VNPP433-3β (433) or Gal (parental compound) for 4 h. DMSO served as control. The cells were harvested, and nuclei isolated. The cytoplasmic and nuclear fractions were immunoblotted for f-AR. α-tubulin served as cytoplasmic loading control and Histone H3 (H.H3*) as nuclear loading control. Nuclear translocation assay was optimized for treatment duration (4 h) to avoid significant degradation of f-AR by VNPP433-3β (433). (**F**) RNA sequencing and subsequent GSEA plot of the VNPP433-3β (433)-treated CWR22Rv1 cells show inhibition of androgen response. The uncropped blots are shown in Figure S1.

Altered androgen signaling contributes significantly to PCa development and progression. Studies showed that activation of the AR suppresses eIF4E phosphorylation whereas antagonizing AR with anti-androgens stimulated eIF4E phosphorylation. As the degradation of AR triggers the increase in eIF4E phosphorylation by Mnk1/2, combination of AR antagonists and mTOR inhibitors was effective in suppressing tumor growth [20]. In order to evaluate the effect of VNPP433-3β on AR, we examined the levels of full-length AR (f-AR) and its splice-variant AR-V7 in LNCaP (androgen-dependent human prostate adenocarcinoma cells that do not express AR-V7) and CWR-R1 and CWR22Rv1 (castration-resistant, androgen-independent human prostate cancer cell lines that express AR-V7) following treatment with varying concentrations of VNPP433-3β (0.6, 1.25, 2.5, 5, 10, 15, 20 µM) for 72 h. Interestingly, we observed that VNPP433-3β promotes significant degradation of f-AR and AR-V7 at 1.25 µM and upwards in all the three cell lines in a dose-dependent manner (Figure 1A–D). Evidently, VNPP433-3β promotes robust degradation of f-AR/AR-V7 besides depleting Mnk1/2 that results in diminution of peIF4E thus inhibiting Mnk1/2-eIF4E oncogenic signaling [13].

### 3.2. Degradation of f-AR by VNPP433-3β Leads to Its Decreased Nuclear Translocation

The f-AR belongs to the steroid receptor superfamily and is a ligand-dependent transcription factor. In the absence of androgens (the ligand), f-AR is localized largely in the cytoplasm. However, upon activation by binding of androgen ligands, f-AR is translocated to the nucleus where it mediates active transcription of the target genes, the androgen-responsive genes. But, in CRPC cells, f-AR remains active in the nucleus and mediates transcription of androgen-responsive genes even in the absence of androgens leading to tumor progression [17,21]. Therefore, sequestration of f-AR in the cytoplasm and its nuclear translocation are key mechanisms of regulating its activity. Since f-AR activity is higher in prostate cancer cells, we examined the effect of VNPP433-3β in nuclear translocation of f-AR. We observed that, treating hormone-starved CWR22Rv1 cells with natural androgen, DHT (10 nM) enhanced the nuclear translocation of f-AR, whereas 10 μM VNPP433-3β or Gal (parental compound) considerably decreased its nuclear translocation as the nuclear fraction showed low levels of f-AR compared to the control and cytoplasmic fractions (Figure 1E). Nuclear translocation assay was optimized for treatment duration (4 h) to avoid significant degradation of f-AR by VNPP433-3β. Consequently, this resulted in decreased transcription of AR target genes, which is further confirmed by RNA sequencing and GSEA analysis wherein the androgen response is found inhibited by VNPP433-3β (433) (Figure 1F).

### 3.3. Decrease in f-AR/AR-V7 by VNPP433-3β Is Due to Enhanced Proteasomal Degradation

**Figure 2:**
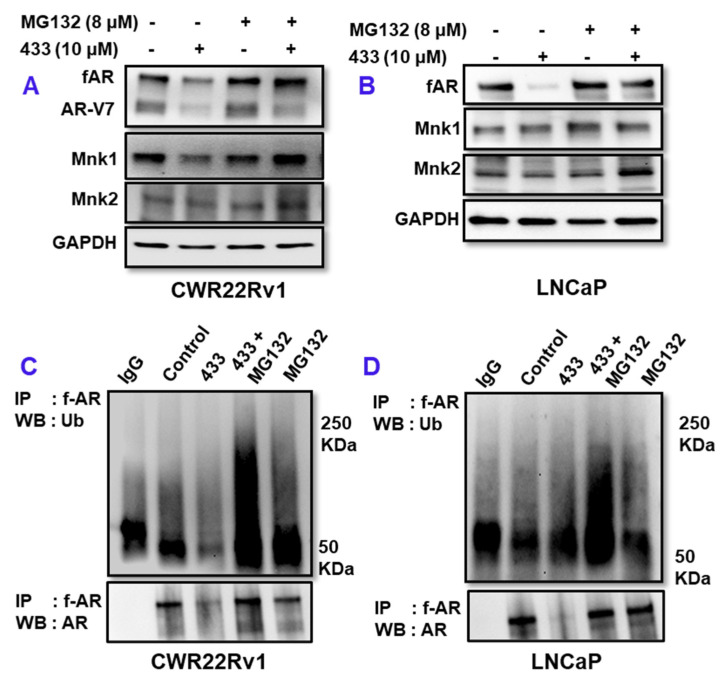
VNPP433-3β enhances ubiquitin-dependent proteasomal degradation of f-AR and AR-V7. (**A**,**B**) Androgen deprived CWR22Rv1 and LNCaP cells were treated with DMSO or VNPP433-3β for 4 h. Cells were then incubated in presence or absence of MG132 (8 μM) for 8 h and immunoblotted for AR, AR-V7, Mnk1 and Mnk2. GAPDH served as loading control. (**C**,**D**) 22Rv1 and LNCaP cells were treated as in (**A**,**B**) and 1 mg of total cell lysate was used for immunoprecipitation and the blots were probed with ubiquitin antibody. The uncropped blots are shown in Figure S2.

We next investigated the effect of VNPP433-3β in proteasomal degradation of AR. MG132 is a potent proteasome inhibitor that decreases degradation of ubiquitinated proteins by the 26 S proteosome in mammalian cells. To study the role of 26S proteasome in VNPP433-3β-induced f-AR/AR-V7 degradation, we treated 22Rv1 or LNCaP cells with 10 µM VNPP433-3β for 4 h followed by MG132 for 8 h. Interestingly, treatment with MG132 abated VNPP433-3β-induced degradation of AR, leading to the accumulation of AR in 22Rv1 (Figure 2A) and LNCaP cells (Figure 2B). A similar observation was found for Mnk1/2 proteins, which are additional targets of VNPP433-3β. The results are exciting as it confirms the proteasomal degradation of AR and its level in the VNPP433-3β + MG132-treated cells was on par with the control cells. All proteins destined for proteasomal degradation are first ubiquitinated by one of the many E3 ligases [22]. Since ubiquitination is a pre-requisite for degradation by 26S proteasome, we next examined the effect of VNPP433-3β in ubiquitination of fAR/AR-V7, if any by immunoprecipitating AR and probing the western blots with ubiquitin antibody. As anticipated, the level of ubiquitinated AR was far higher in the cells treated with VNPP433-3β than the control in CWR22Rv1 (Figure 2C) and LNCaP cells (Figure 2D). These results strongly suggest that VNPP433-3β induces degradation of f-AR via E3 ligase ubiquitin-proteasomal pathway.

### 3.4. Real Time Quantitative Assay of VNPP433-3β-Induced AR Degradation in Live CWR22Rv1 Cells

**Figure 3:**
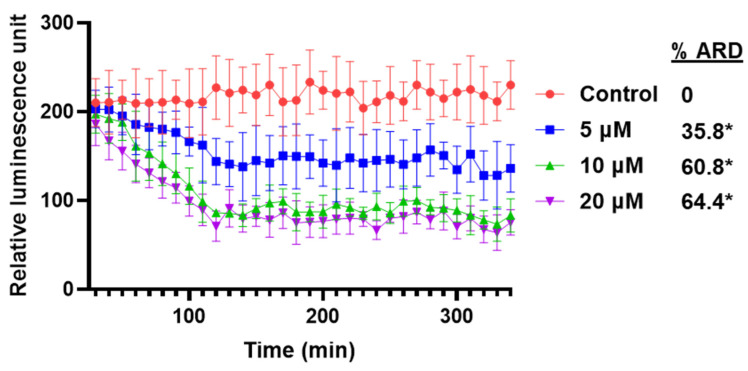
VNPP433-3β-mediated degradation of AR occurs rapidly and is concentration- and time-dependent. CRISPR-edited 22Rv1 cells that express endogenous AR labeled with HiBiT and LgBiT were treated with 5, 10 and 20 µM of VNPP433-3β in presence of luminescence substrate Nano-Glo^®^ Endurazine™. Luminescence was measured every 10 min for 5 h and plotted against time using Graphpad Prism software. %ARD = % androgen receptor (f-AR) degradation following treatments with increasing concentrations of VNPP433-3β. * *p* < 0.0001.

Using different biochemical approaches, we have demonstrated that VNPP433-3β enhances proteasomal degradation of AR thereby significantly decreasing its level in PCa cells. To further validate this finding and kinetic studies of AR degradation in real time in live cells, we employed CRISPR-edited CWR22Rv1 cells that express endogenous AR labeled with HiBiT, a small 11 amino acid peptide that binds with high affinity to another larger subunit LgBiT. LgBiT is a luciferase that is non-functional; but binding HiBiT renders it functional due to complementation. When the HiBiT cells are transfected with LgBiT expression vector, the protein forms a functional complex (HiBiT-LgBiT complex) that can produce luminescence in the presence of suitable substrate. The luminescence is proportional to the amount of AR in the PCa cell [23]. Interestingly, the level of AR reflected by RLU (Relative Luminescence Unit) started decreasing in 30 min after addition of VNPP433-3β in all tested concentrations. The luminescence steeply declined until 2 h thereafter continued to decline slowly. The level of AR was significantly decreased by 35.8%, 60.8% and 64.4% in 5 µM, 10 µM and 20 µM VNPP433-3β treatment respectively (Figure 3).

### 3.5. VNPP433-3β Acts as Molecular Glue between AR and MDM2

**Figure 4:**
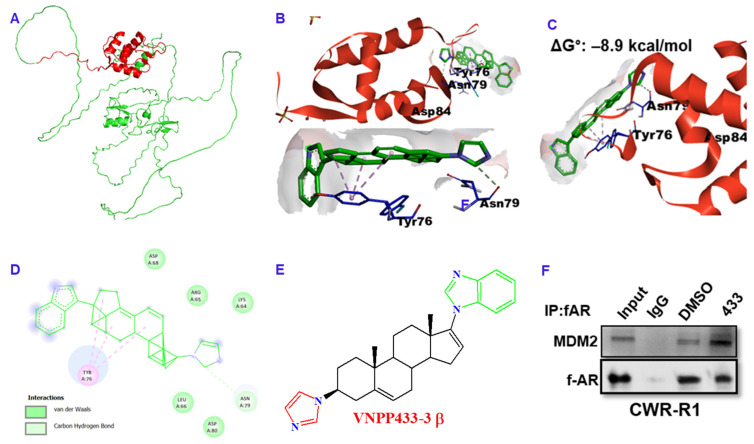
VNPP433-3β acts as molecular glue that induces proximity in MDM2 and f-AR, facilitating MDM2-mediated ubiquitination of f-AR. (**A**) Predicted structure of full-length MDM2 (Alpha fold). Region in red represents amino terminal domain (amino acids 17–125) used for docking. (**B**) X-ray crystallographic structure of amino terminal domain (amino acids 17–125, PDB ID 2AXI) showing VNPP433-3β docked on protein surface with high affinity; ΔG°: –8.9 kcal/mol. (**C**) The ligand is bound to the surface of MDM2 primarily through the amino acids Tyr 76, Asn 79 and Asp 84, leaving a large portion of the ligand exposed and available to bind AR. (**D**) Major interactions (pi-alkyl with Tyr 76 and Carbon-Hydrogen boding with Asn 79) of VNPP433-3β with protein peripheral surface amino acids. (**E**) The 2D chemical structure of VNPP433-3β. (**F**) Co-immunoprecipitation of f-AR and MDM2 demonstrates that interaction of f-AR and MDM2 is enhanced in presence of VNPP433-3β. The CWR-R1 is an androgen-independent cell line derived from CWR22 tumors that expresses functional mutated AR (H874Y) and more accurately represent genetic composition of recurrent tumors than the immortalized DU145 or PC3 cell lines [28]. CWR-R1 cells were hormone-starved for 48 h and treated with DMSO or VNPP433-3β for 4 h. Cell lysate containing 1 mg protein was subjected to immunoprecipitation and subsequent immunoblotting for f-AR and MDM2. The uncropped blots are shown in Figure S2.

Several studies show that physiologically, AR is regulated partly by MDM2-mediated ubiquitination and subsequent proteasomal degradation [24,25,26]. Since AR binds VNPP433-3β with high affinity (ΔG° = –8.3 kcal/mol) [13], we further examined the affinity of VNPP433-3β for MDM2 using molecular modeling. Figure 4A presents the predicted structure of full-length MDM2 (Alphafold). X-ray crystallographic structure of N-terminal domain [27] that interacts with the client proteins for ubiquitination is used for docking with VNPP433-3β. Interestingly, it was found that VNPP433-3β interacts with the amino acids located on the peripheral surface of MDM2 with high affinity (ΔG° = –8.9 kcal/mol, Figure 4B–D). As shown in the model, much of the molecular surface of VNPP433-3β is exposed and available to interact with AR. The potential interaction predicted by molecular modeling was further confirmed by co-immunoprecipitation of fAR and MDM2 in CWR-R1 cells treated with VNPP433-3β, wherein it enhanced the interaction (Figure 4E).

### 3.6. RNA Sequencing Shows Transcriptional Downregulation of Several AR-Target Genes

**Figure 5:**
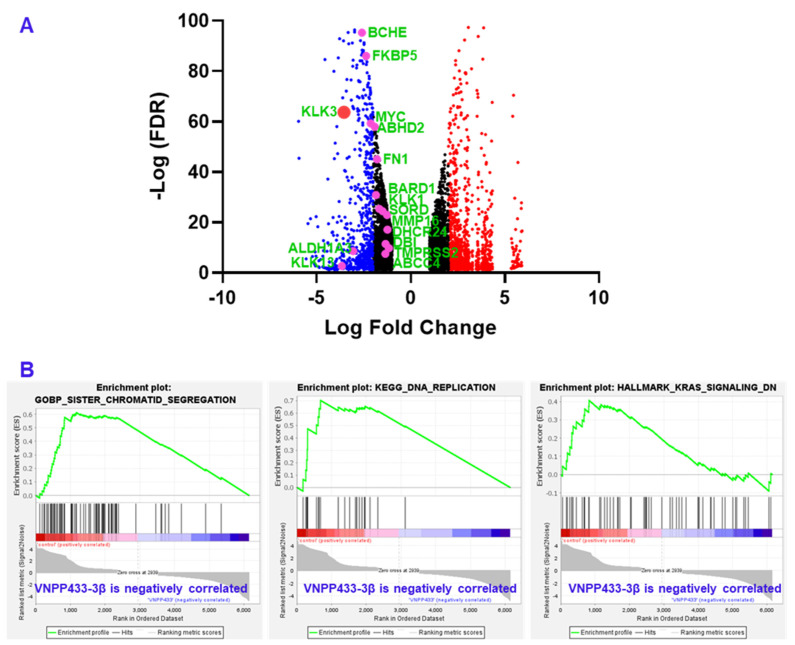
RNA sequencing and gene expression analysis shows that several androgen-responsive genes that are transcriptional target of AR are significantly downregulated including KLK3 that encodes prostate specific antigen (PSA). (**A**) Volcano plot of differentially expressed genes upon treating CWR22Rv1 cells with VNPP433-3β for 24 h. Key AR target genes are highlighted as pink dots labeled in green and presented in Table 1. Prostate specific antigen (PSA) or KLK3 is represented as large red dot. Blue and small red dots indicate downregulated and upregulated genes respectively. Black dots represent genes that are down or upregulated less than two LFC. (**B**) The Gene Set Enrichment Analysis (GSEA) plot showing inhibition of pathways (sister chromatid segregation, DNA replication and KRAS signaling) critical to prostate cancer development and progression.

Since AR is a known transcriptional factor that mediates the transcription of AR-responsive genes, we next evaluated the transcriptional variation of several key target genes by RNA sequencing. Various studies have identified several genes that are transcriptional targets of AR. We specifically analyzed major genes that appear in more than five independent studies as targets of AR as reviewed [29]. All genes in the list that were manifested in the RNA sequencing data of VNPP433-3β-treated 22Rv1 cells were found to be downregulated as shown in Figure 5A. The genes, their physiological function and role in tumorigenesis is presented in Table 1. Remarkably, KLK3 gene that encodes prostate specific antigen (PSA) was downregulated 11.7 times compared to DMSO control. Further, the gene set enrichment analysis (GSEA) of RNA sequencing data shows that genes of several pathways critical for prostate cancer progression including DNA replication, sister chromatid segregation and KRAS signaling are downregulated by VNPP433-3β (Figure 5B).

## 4. Discussion

**Figure 6:**
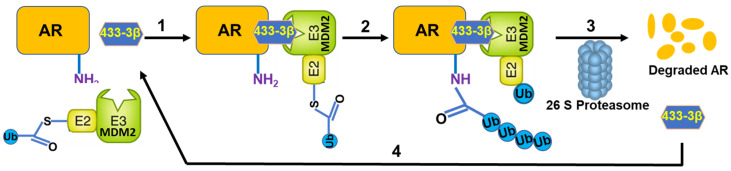
Schematic of the VNPP433-3β (molecular glue)-induced androgen receptor (f-AR) ubiquitin-proteasome degradation. A ternary complex formed upon binding of VNPP433-3β (433-3β, molecular glue), target protein (f-AR) and MDM2 E3 ligase complex (step 1), to promote f-AR protein ubiquitination (Ub) (step 2), followed by f-AR degradation by the proteasome plus release of VNPP433-3β (step 3). Free VNPP433-3β can then bind another molecule of AR (step 4) to repeat the degradation cycle.

Nearly all the natural and synthetic ‘molecular glue degrader’ small molecules are discovered serendipitously while the PROTACS are rationally designed and developed [4]. Earlier, we had reported the potential of VNPP433-3β in treating preclinical models of castration-resistant prostate cancer by promoting degradation of f-AR and preventing phosphorylation of eIF4E by depleting Mnk1/2 [11,30]. VNPP433-3β is rigorously tested for host toxicity and pharmacokinetics in mice and rat and found that it is well-tolerated at the concentrations used for in vivo anti-tumor efficacy studies [31]. In this study, we demonstrate the potential role of VNPP433-3β as molecular glue in promoting targeted degradation of f-AR in prostate cancer cells by induced proximity with the E3 ligase MDM2. Physiologically in normal prostate cells, f-AR is degraded by MDM2-mediated ubiquitination and subsequent proteasomal degradation [11,31]. Interestingly, VNPP433-3β significantly decreased cytoplasmic f-AR and limited its nuclear translocation even in presence of DHT, the natural ligand of f-AR. Since f-AR is a ligand-regulated transcription factor and normally localized in cytoplasm, its translocation to nucleus upon activation by androgen-binding is crucial for its function as transcriptional factor. Molecular modeling and co-immunoprecipitation suggest the role of VNPP433-3β as molecular glue and chemically inducing proximity of f-AR and E3 ligase MDM2, apparently by interacting with both proteins and stabilizing the ternary complex. Treating the prostate cancer cells with proteasome inhibitor MG132 averted VNPP433-3β-induced degradation of AR but lead to accumulation of ubiquitinated f-AR and AR-V7, potentially due to f-AR hetero-dimerization (f-AR and AR-V7) as previously described [32]. This confirms the involvement of VNPP433-3β in promoting ubiquitin-mediated proteasomal degradation of f-AR and AR-V7 by enhancing its interaction with E3 ligase MDM2. Furthermore, HiBiT-LgBiT reporter assay is robust and combines the power of CRISPR-mediated gene editing and bioluminescent reporter system of tagged proteins. As the f-AR is specifically tagged with HiBiT, the method is relatively infallible compared to any other methods in use currently [19]. Based on these results, the proposed mechanism of VNPP433-3β-induced f-AR degradation is depicted in Figure 6. Thus, VNPP433-3β (the molecular glue) induces the formation of a ternary complex, in which MDM2 E3 ligase bound to an E2—Ub ubiquitinates f-AR protein, causing the subsequent f-AR degradation by the proteasome plus release of VNPP433-3β. Free VNPP433-3β can then bind another molecule of f-AR to repeat the degradation cycle.

f-AR is known to activate transcription of several genes, the androgen-responsive genes. Failure of f-AR translocation into the nucleus is further reflected as decreased transcription of f-AR target genes as revealed by transcriptome profiling. Nearly all prostate cancer patients express elevated levels of prostate specific antigen (PSA) encoded by the gene KLK3 and is often considered as indicative marker of prostate cancer and or anomalous functioning of the prostate gland [33]. RNA sequencing reveals that KLK3 (PSA) and other transcriptional target genes of AR are significantly downregulated upon VNPP433-3β treatment besides inhibiting transcription of several genes in pathways critical for prostate cancer progression in prostate cancer cells. Our findings reveal the role of VNPP433-3β as molecular glue that chemically induce proximity of f-AR and MDM2, thereby facilitating the ubiquitination of f-AR by MDM2 and its subsequent proteasomal degradation relevant to pharmacological inhibition of prostate cancer tumor growth via inhibition of cell proliferation and induction of apoptosis.

## 5. Conclusions

VNPP433-3β inhibits nuclear translocation of f-AR and f-AR-mediated oncogenic transcription by chemically inducing proximity of f-AR and E3 ligase MDM2 triggering targeted degradation of f-AR rapidly thereby inhibiting development and progression of prostate cancer. Because targeted protein degradation has a few putative advantages, including the simultaneous destruction of the target’s scaffold and enzymatic/antagonistic activities, and the ability to catalytically turn over the target protein within a cell, the development of VNPP433-3β as a potential therapeutic for prostate cancer is well justified.

## Figures and Tables

**Table 1:** The genes that are transcriptional targets of AR [29] found to be downregulated upon VNPP433-3β treatment as revealed by RNA sequencing. All genes except NKX3-1 play crucial role in tumorigenesis.

Gene	LFC	−Log (FDR)	Remarks
KLK3	−3.549878878	63.74105	Prostate specific antigen (PSA)
KLK1	−1.499349751	24.47005	Serine protease; Oncoprotein
KLK13	−3.661495531	2.685301	Serine protease; Oncoprotein
NKX3-1	−2.987220702	106.1704	Transcription factor in prostate epithelium, tumor suppressor
DHCR24	−1.242758268	17.15381	Flavin adenine dinucleotide-dependent oxidoreductase; cholesterol biosynthesis
DBI	−1.340163994	11.44401	acyl-CoA binding protein, cell signaling, steroid synthesis
MMP16	−1.264127518	22.95686	Cancer metastasis, matrix metalloproteinase
FKBP5	−2.367045284	86.07973	Co-chaperone that regulates steroid hormone receptors, immunoregulation
ALDH1A3	−3.05609345	8.603198	Tumorigenesis, detoxification of aldehydes and lipid peroxidation
BCHE	−2.590390622	95.29679	Nonspecific cholinesterase
ABHD2	−1.915809808	57.9697	Enables nuclear steroid receptor activity, highly expressed in spermatozoa
SORD	−1.673855387	25.39808	Carbohydrate metabolism
ABCC4	−1.351567948	7.512045	Multidrug resistance, regulates intracellular yclic nucleotide levels, cAMP-dependent signaling to the nucleus
BARD1	−1.850384403	30.88807	DNA repair, E3 ubiquitin ligase, transcriptional regulation to maintain genomic stability
TMPRSS2	−1.160624783	9.802972	Transmembrane Serine Protease 2, upregulated by androgenic hormones in prostate cancer, facilitates virus entry
FN1	−1.795774135	45.01869	Component of extracellular matrix, upregulated in multiple cancers
MYC	−2.129324177	59.28253	Oncoprotein

## Data Availability

The datasets used and analyzed during the current study are available from the corresponding author on reasonable request.

## Supplementary Materials

The following supporting information can be downloaded at: https://www.mdpi.com/article/10.3390/cancers15041198/s1, Figure S1: The uncropped blots of Figure 1; Figure S2: The uncropped blots of Figure 2 and Figure 4F.
