# Supplementary material for: Targeted Degradation of Androgen Receptor by VNPP433-3β in Castration-Resistant Prostate Cancer Cells Implicates Interaction with E3 Ligase MDM2 Resulting in Ubiquitin-Proteasomal Degradation
Source: Cancers (Basel). 2023 Feb 14;15(4):1198. doi: 10.3390/cancers15041198 (PMC9954018; doi:10.3390/cancers15041198)
Supplement: Supplementary file 1 [file cancers-15-01198-s001.zip › cancers-2172358-supplementary.pdf]

Targeted Degradation of Androgen Receptor by VNPP433-3β in Castration-Resistant Prostate Cancer Cells Implicates Interaction with E3 Ligase MDM2 Resulting in Ubiquitin-Proteasomal Degradation

Elizabeth Thomas*[1,2†], Retheesh S. Thankan[1,2,4,†], Puranik Purushottamachar[1,2], David J. Weber[5,6,7] and Vincent C. O. Njar*[1,2,3]

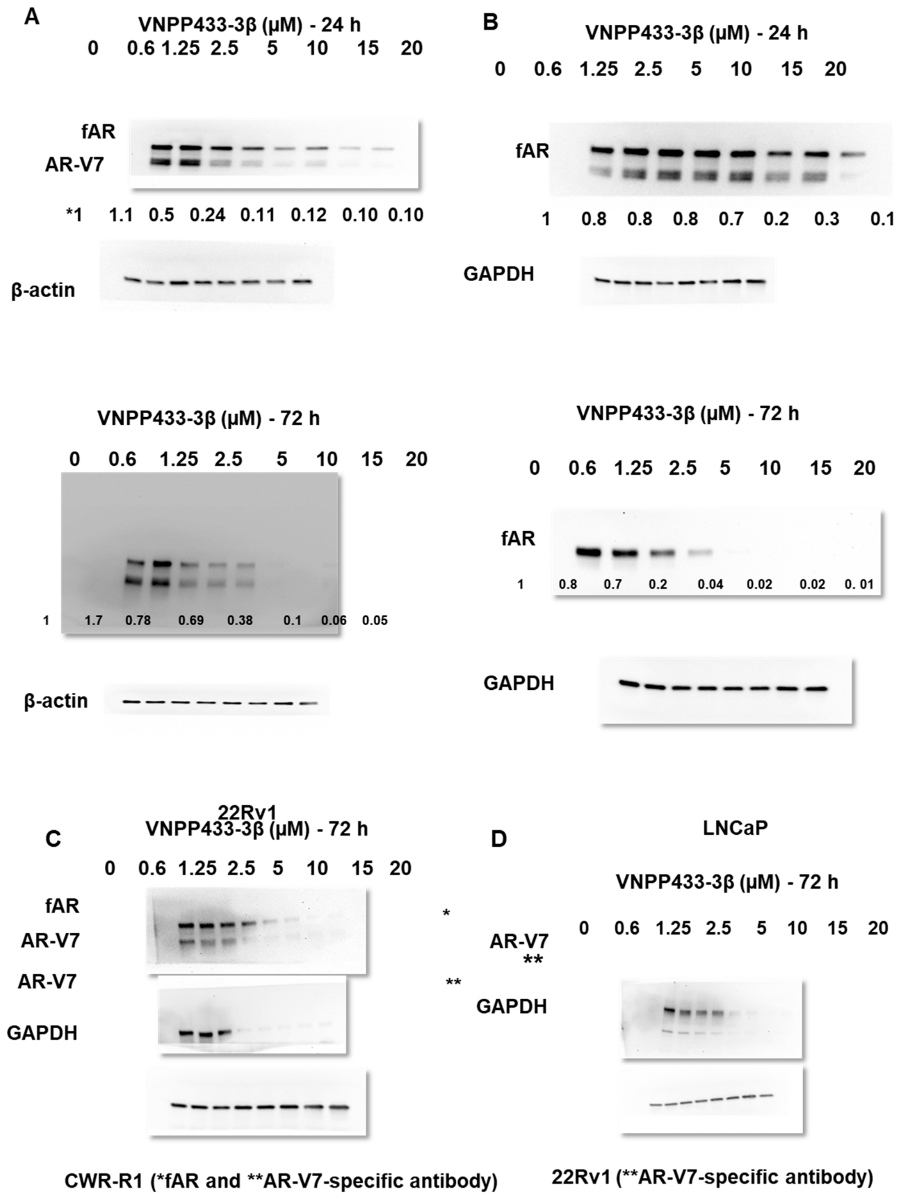

Figure S1. The uncropped blots of Figure 1.

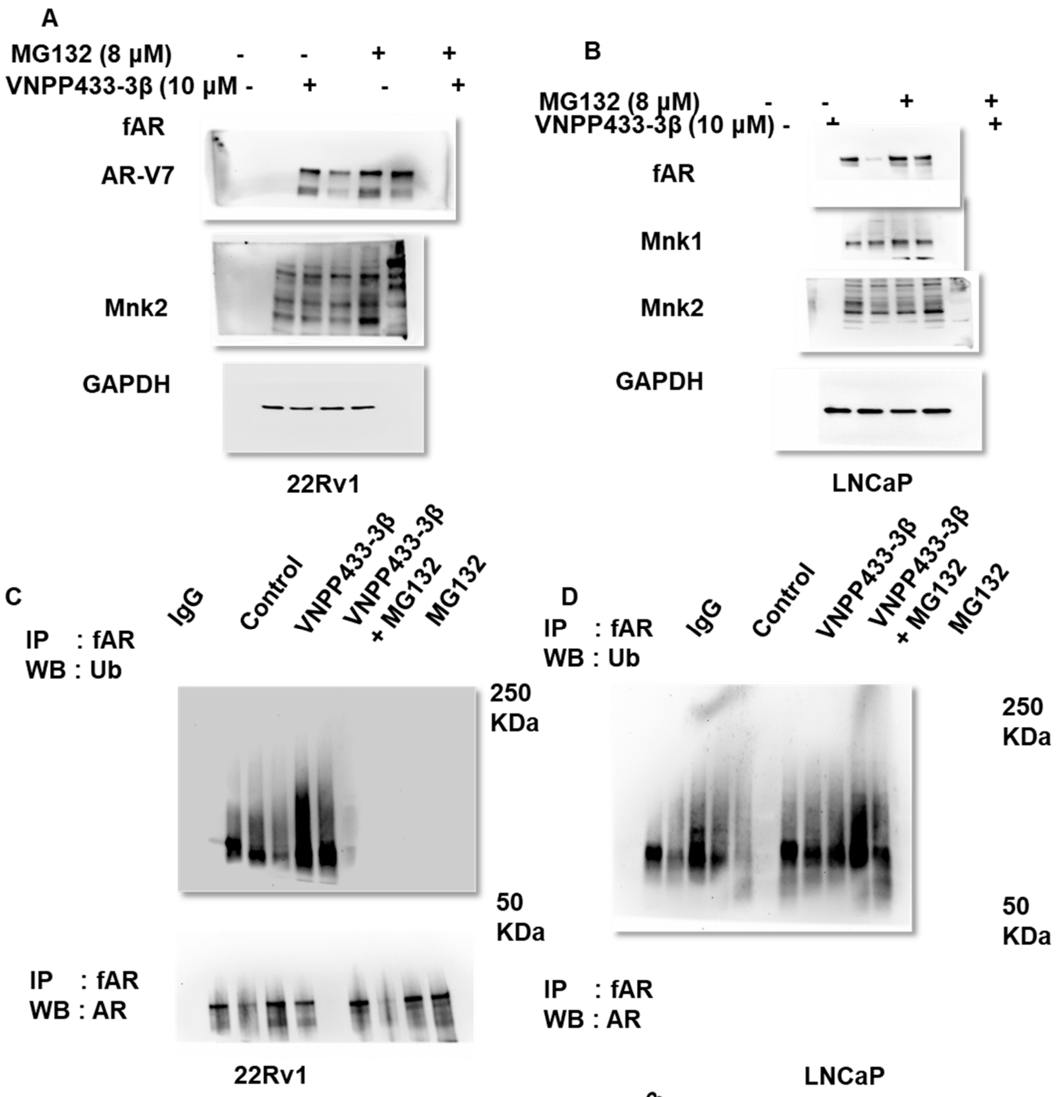

Figure 4 F

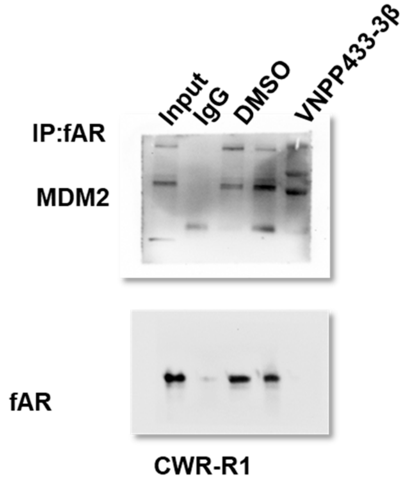

Figure S2. The uncropped blots of Figures 2 and 4F.
